# Treatment Options for Brain Metastases

**DOI:** 10.1007/s11864-024-01195-3

**Published:** 2024-07-22

**Authors:** Ariel Choi, John Hunting, Claire Lanier, Emily Douglas, Pierre Triozzi, Jimmy Ruiz, Marc Benayoun, Jaclyn White, Michael Chan

**Affiliations:** 1 Department of Radiation Oncology, Wake Forest School of Medicine, Winston-Salem, NC USA; 2 Department of Medicine (Hematology and Oncology), Wake Forest School of Medicine, Winston-Salem, NC USA; 3 Department of Radiology at Wake Forest School of Medicine, Winston‑Salem, NC USA; 4 Department of Neurosurgery, Wake Forest School of Medicine, Winston-Salem, NC USA

**Keywords:** Brain metastases, Stereotactic radiosurgery, Whole brain radiation

## Abstract

Therapies for brain metastasis continue to evolve as the life expectancies for patients have continued to prolong. Novel advances include the use of improved technology for radiation delivery, surgical guidance, and response assessment, along with systemic therapies that can pass through the blood brain barrier. With increasing complexity of treatments and the increased need for salvage treatments, multi-disciplinary management has become significantly more important.

## Introduction

There are nearly 200,000 patients diagnosed with brain metastases in the US each year, making this one of the larger subsets of the cancer population [1]. Over the past three decades, there has been a stage migration of brain metastases from a population of large metastases that present due to symptoms to the present population consisting mainly of small occult lesions that are discovered due to screening during the staging process [2]. Brain metastasis management has evolved with this migration along with the publication of multiple randomized trials that have helped define the roles of surgery and stereotactic radiosurgery (SRS). Due to improvements in systemic therapies for multiple cancer histologies, survival in the brain metastasis population has improved over time [3]. As such, goals of management have evolved to include cognitive preservation, distinguishing treatment effects from progressive disease, and management of what can sometimes be multiple serial recurrences. Systemic therapies that cross the blood brain barrier as well as novel technologies such as hippocampal avoidant whole brain radiation (WBRT) and laser interstitial thermotherapy have begun carving out important roles in brain metastasis management. While the proportion of patients that die of brain metastases has decreased over time, there remains a population in the modern setting that will continue to experience neurologic death. The goal of the present review is to summarize some of the modern treatment options available to patients for treatment of brain metastases.

## Diet and lifestyle

There is emerging data that brain metastasis patients with lung cancer primaries may benefit from smoking cessation [4]. While this data remains to be validated, smoking cessation can have other health benefits in the lung cancer population.

## Pharmacologic treatment

### Treatment or prevention of radiation toxicity

Pharmacologic options for mitigation of radiation-induced brain toxicity have improved over time. Dexamethasone has been used for the management of vasogenic edema for decades. It can also be used to treat edema resulting from radiation necrosis. Unfortunately, in cases of severe radiation necrosis, patients may require steroids for prolonged periods or even become steroid dependent. In such cases, patients ultimately experience the toxicities of prolonged steroid usage such as immunosuppression, myopathy, irritability, and osteopenia. In cases of more significant radiation necrosis, bevacizumab has emerged as a non-surgical option to treat radiation necrosis. Two randomized trials have been attempted thus far to assess for bevacizumab’s efficacy in the treatment of symptomatic radiation necrosis. The first trial demonstrated a 100% response rate to bevacizumab in its ability to decrease edema. The second trial, comparing bevacizumab to steroids, closed early and has not presented results thus far.

Radiation-induced cognitive decline (RICD), another toxicity of brain radiotherapy, has been a growing concern for patients and providers as patients with brain metastases are living longer than they had in the past. Cognitive toxicities of radiotherapy do not resolve with time, though they can worsen. Several trials have been performed assessing pharmacologic agents that may have a role in mitigating the likelihood and severity of RICD. The RTOG 0614 is thus far the only phase III trial demonstrating a benefit of a pharmacologic agent (in this case the addition of memantine to whole brain radiation) in terms of the likelihood of cognitive decline.

Dexamethasone: 1–16 mg daily (titrated to relief of neurologic symptoms).

Contraindications: hypersensitivity, systemic fungal infection, diabetes, uncontrolled infection, osteoporosis, cirrhosis, renal insufficiency, myasthenia gravis, ulcerative colitis, peptic ulcer disease.

Drug interactions: cytochrome p450 inhibitors.

Main side effects: insomnia, hyperglycemia, rash, reflux, increased risk of infection, fluid retention, weight gain, increased appetite, nausea, vomiting, agitation, hepatotoxicity, myopathy, avascular necrosis.

Special Points: This corticosteroid is the first-line medication used to manage vasogenic cerebral edema due to tumor-related disruption of the blood–brain barrier [5]. Due to its numerous acute and chronic side effects, it should be tapered in an individualized manner weighing the patient’s neurologic symptoms vs. steroid-related adverse effects. It is generally not given concurrently with immunotherapy, given the presumption that it may reduce the efficacy of antitumor immunity with its immunosuppressive effects; however, this is not definitively borne out by research studies and remains somewhat unclear [6].

Bevacizumab 5 mg/kg once every 2 weeks.

Contraindications: hypersensitivity, surgical procedure within 4 weeks, bleeding diathesis, coagulopathy, acute intracranial hemorrhage.

Drug interactions: NSAIDs, anticoagulants, anti-platelet agents, corticosteroids.

Main side effects: hypertension, proteinuria, hemorrhage, gastrointestinal perforation (rare but serious).

Special points: This drug can be used to treat radiation necrosis caused by prior SRS [7]. It may provide advantages over surgical intervention when radiation necrosis occurs in the eloquent brain.

Memantine 10 mg twice daily (starting dose at 5 mg once daily with an escalation of 5 mg/day each week until reaching the full dose).

Contraindications: hypersensitivity, seizure disorder, severe cardiac, renal or hepatic dysfunction.

Drug interactions: NMDA antagonists, alkalinizing agents, carbonic anhydrase inhibitors.

Main side effects: headache, dizziness, confusion.

Special points: This drug may be taken in conjunction with whole brain radiotherapy or hippocampal avoidant whole brain radiotherapy to mitigate the cognitive toxicities caused by the radiotherapy [8].

### Lung cancer

The presence of the blood brain barrier(BBB) prevents larger molecules and water-soluble substances from passing into the brain. This has prevented cytotoxic chemotherapies and many targeted agents from reaching brain metastases, leaving surgery and radiotherapy as the first-line options for treatment of brain metastases. However, second- and third-generation tyrosine kinase inhibitors (TKI) have been developed recently that can cross the BBB due to small size and lipophilic nature. Moreover, they have been engineered to avoid binding to efflux transporters in the BBB in order to maintain CNS concentration. Trials have demonstrated that for patients with NSCLC that harbor such mutations as EGFR and ALK, these newer generation TKI may be used as first-line therapy.

Osimertinib 160 mg once daily.

Contraindications: hypersensitivity.

Drug interactions: QT-prolonging agents, aminosalicylic acid.

Main side effects: rash, nail disease, anemia, lymphopenia, thrombocytopenia, fatigue, interstitial lung disease (rare but serious).

Special points: This drug is first-line therapy for patients with brain metastases from metastatic lung adenocarcinoma with EGFR exon 19 deletion or exon 21 L858R mutation if patients have not developed brain metastases while on this drug [9•]. Patients may be treated in the absence of radiation therapy if there is no evidence of severe mass effect or impending herniation.

Alectinib 600 mg twice daily.

Contraindications: hypersensitivity.

Drug interactions: bradycardia-inducing agents.

Main side effects: rash, bradycardia, constipation, anemia, hyperbilirubinemia, AST/ALT elevation, fatigue, decreased renal function.

Special points: This drug is first-line therapy for patients with brain metastases from metastatic lung adenocarcinoma with ALK translocation if patients have not developed brain metastases while on this drug [10]. Patients may be treated in the absence of radiation therapy if there is no evidence of severe mass effect or impending herniation.

Lorlatinib 100 mg once daily.

Contraindications: hypersensitivity, strong cytochrome p450 inhibitors.

Drug interactions: cytochrome p450 inducers.

Main side effects: peripheral edema, hyperlipidemia, hyperglycemia, anemia, neuropathy, arthralgia, AST/ALT elevation, decreased renal function.

Special points: This drug can be used as second-line therapy for brain metastasis patients with ALK translocation who have had CNS progression on alectinib or other second-generation ALK inhibitors [11]. In this clinical scenario, the intracranial response rate is approximately 50% and thus, treatment may be more optimal if combined with local therapies such as radiation.

### Melanoma

The role of systemic agents in the management of brain metastases from melanoma has also increased over time. Traditionally, brain metastases have been a major cause of death for patients with melanoma due to the combination of intracranial hemorrhage, leptomeningeal spread, and high brain metastasis velocity [12, 13]. Since the advent of novel systemic therapies in the mid-2010s (particularly immune checkpoint inhibitors), patient outcomes have improved, and this improvement appears to have been driven by a decrease likelihood of dying of brain metastases [14]. At the present time, there are still limitations of these systemic agents. For brain metastases to respond well to immunotherapy, dual immunotherapy is generally required. Toxicity from this combination make tolerance an issue. The combination of BRAF and MEK inhibitors can generally only be given to those with a BRAF mutation, and even then, the responses are commonly short lived.

Ipilimumab 3 mg/kg every three weeks with Nivolumab 1 mg/kg every three weeks concurrently with ipilimumab, followed by 3 mg/kg every 2 weeks maintenance.

Contraindications: hypersensitivity, severe or active autoimmune disease, prior organ transplant where graft failure would be life-threatening.

Drug interactions: corticosteroids, acetaminophen, antibiotics, proton pump inhibitors.

Main side effects: dermatitis, endocrinopathies, colitis, hepatitis, nephritis, rash, abdominal pain, diarrhea, anemia, nausea, increased AST/ALT, cough, musculoskeletal pain.

Special points: This combination can be used as first-line therapy for melanoma brain metastases in which patients have been previously been exposed to dual immunotherapy. Of note, patients who have larger or symptomatic lesions may be more optimally treated if some form of local therapy is included [15].

Dabrafenib 150 mg twice daily with Trametinib 2 mg once daily.

Contraindications: hypersensitivity.

Drug interactions: cytochrome p450 inhibitors, QT-prolonging agents, proton pump inhibitors, oral contraceptives, immunosuppressants, statins, warfarin.

Main side effects: rash, cutaneous squamous cell carcinoma, hyperglycemia, headache, arthralgia, fever, hypophosphatemia, edema, hypertension, rash, hypoalbuminuria, diarrhea, anemia, AST/ALT elevation.

Special points: This drug combination can be used as first-line therapy in isolated instances for patients with brain metastases from melanoma. Dabrafenib blocks the BRAF pathway, while Trametinib prevents the reactivation of the BRAF pathway by inhibiting MEK. In patients with BRAF-mutated melanoma and a high burden of both intracranial and extracranial disease, this combination may be a good upfront option. While this combination can be associated with high response rates, the responses tend to be transient [16].

### Breast cancer

The breast cancer population with brain metastases commonly has a longer survival than brain metastasis patients with other primary cancers due to the many effective systemic therapies for metastatic breast cancer. Outcomes from breast cancer brain metastases are closely correlated to receptor status, as patients with Her2 and hormone receptor positivity have better prognosis [17]. However, in spite of prolonged survival, many of these patients will ultimately experience relapse within the brain, and some patients will experience multiple serial recurrences which will require management. The response rate for brain metastases from breast cancer-directed systemic therapies has been suboptimal (generally less than 50%), and therefore these systemic options have been either reserved for salvage therapy or used as a treatment to prevent new and more numerous brain metastases. The majority of available agents at this time are for patients whose tumors overexpress Her2.

Tucatinib 300 mg twice daily.

Contraindications: hypersensitivity.

Drug interactions: cytochrome p450 inhibitors.

Main side effects: diarrhea, rash, hypoalbuminemia, increased AST/ALT, decreased appetite, hepatotoxicity (rare but serious).

Special points: This drug can be used in combination with capecitabine and trastuzumab for her2 positive breast cancer patients with brain metastases. While local therapies such as radiation remain the first line for brain metastases, this regimen can be initiated after local therapy to mitigate the risk of new metastases. The regimen may also be considered as a salvage regimen for resistant brain metastases that have failed multiple local therapies [18•].

Capecitabine 1000 mg/m2 twice daily on days 1–14 of a 3-week cycle.

Contraindications: hypersensitivity, complete absence of dihydropyrimidine dehydrogenase activity, concurrent sorivudine.

Drug interactions: aspirin, QT-prolonging agents, proton pump inhibitors, folic acid, leucovorin, warfarin, enoxaparin.

Main side effects: hand foot syndrome, diarrhea, stomatitis, decreased appetite, fatigue, cardiac event (rare but serious), severe bone marrow suppression (rare but serious).

Special points: This drug can be used in combination with blood brain barrier penetrating Her2 inhibitors to mitigate the risk of new brain metastases after first-line local therapy or as salvage therapy for resistant breast cancer brain metastases that have failed multiple local therapies.

Neratinib 240 mg daily.

Contraindications: hypersensitivity.

Drug interactions: proton pump inhibitors, H2 blockers, cytochrome p450 inhibitors.

Main side effects: diarrhea, abdominal pain, fatigue, rash.

Special points: This drug can be used in combination with capecitabine as salvage therapy for patients who have Her2 positive breast cancer brain metastases that have progressed beyond tucatinib-based therapy [19].

Lapatinib 1250 mg daily.

Contraindications: hypersensitivity, strong cytochrome p450 inhibitors.

Drug interactions: QT-prolonging agents, cytochrome p450 inhibitors, dexamethasone.

Main side effects: diarrhea, increased AST/ALT, reversible myocardial toxicity, prolonged QT interval, interstitial lung disease (rare but serious), hepatotoxicity (rare but serious).

Special points: This drug can be used in combination with capecitabine as later-line therapy for patients who have Her2 positive breast cancer brain metastases that have progressed beyond early-line therapies [20].

Trastuzumab deruxtecan 5.4 mg/kg once every 3 weeks.

Contraindications: hypersensitivity.

Drug interactions: cytochrome p450 inhibitors.

Main side effects: nausea, vomiting, constipation, stomatitis, pneumonitis, reversible myocardial toxicity, neutropenia (rare but serious).

Special points: Early studies suggest that there is modest CNS activity for this agent for Her2 positive breast cancer brain metastases. As such, it may be a reasonable option for breast cancer brain metastasis patients that have progressed beyond a capecitabine-containing regimen [21].

Trastuzumab-emtansine 3.6 mg/kg IV every 3 weeks.

Contraindications: hypersensitivity.

Drug interactions: strong cytochrome p450 inhibitors.

Main side effects: thrombocytopenia, peripheral neuropathy, increased AST/ALT, hepatotoxicity (rare but serious), cardiotoxicity.

Special points: This drug can be used for patients with HER2 positive breast cancer and brain metastases that have progressed beyond tucatinib-based therapy [22].

Abemaciclib 150 mg by mouth twice daily.

Contraindications: hypersensitivity.

Drug Interactions: cytochrome p450 inhibitors.

Main side effects: diarrhea, myelosuppression, nausea, increased AST/ALT.

Special points: This drug alone, or in combination with endocrine therapy, has shown modest intracranial clinical benefit in patients with metastatic estrogen-receptor positive, HER2 negative breast cancer with brain metastases [23].

## Interventional procedures

Radiation therapy has evolved over time for treatment of brain metastases. Several randomized trials have now demonstrated that for patients with 4 or fewer brain metastases, SRS leads to improved cognitive outcomes over WBRT [24–27]. WBRT tends to be reserved for clinical situations of more numerous lesions. At present, trials are being performed to determine what is the maximum number of lesions in which SRS is the appropriate option [28]. For patients who require WBRT, hippocampal avoidant(HA-) WBRT has recently demonstrated improved cognitive outcomes over traditional WBRT [29•]. Advancements in technology such as linear accelerator-based SRS and single isocenter multiple target (SIMT) SRS have made the ability to perform radiosurgery ubiquitous [1].

As practitioners have become more aggressive with non-surgical approaches for brain metastases, hypofractionation has become more favored in the treatment of metastases with higher risk of post-treatment toxicity such as larger tumors [30] and tumors treated in patients receiving immunotherapy [31]. Presently trials are being performed to determine if this approach improves the therapeutic ratio otherwise provided by single fraction SRS.

### Stereotactic radiosurgery (SRS)

Contraindications: lesions greater than 3 cm, diffuse leptomeningeal disease.

Complications: treatment-related edema, radiation necrosis.

Special points: Randomized trials have demonstrated improved cognitive function for patients with 4 or fewer brain metastases with SRS [25]. Treating greater than 4 lesions has been demonstrated to be feasible and effective, though these patients have greater distant brain failure [32], higher brain metastasis velocity [33] and greater need for salvage therapy. SRS can also be used in the post-operative [34] and pre-operative [35] setting. Data is emerging for efficacy of SRS in patients with small cell lung cancer [36•].

### Hypofractionated stereotactic radiotherapy (hfSRT)

Contraindications: lesions greater than 5 cm.

Complications: treatment-related edema, radiation necrosis.

Special points: This treatment can be used to treat lesions that are larger than what can traditionally be safely treated with single fraction SRS [30]. Hypofractionation can also be used in a setting of brain metastases that have previously received SRS to the same lesion [37].

### Whole brain radiotherapy (WBRT)

Contraindications: prior WBRT, severe dementia, concurrent cytotoxic chemotherapy.

Complications: radiation-induced cognitive decline, decline in performance status.

Special points: This treatment is more appropriate for patients with numerous brain metastases and leptomeningeal involvement. A recent study brought into question the possible benefit of WBRT in patients with asymptomatic brain metastases when the performance status is poor or the life expectancy is limited [38].

### Hippocampal-avoidant whole brain radiotherapy (HA-WBRT)

Contraindications: prior WBRT, concurrent cytotoxic chemotherapy.

Complications: radiation-induced cognitive decline, decline in performance status.

Special points: A randomized trial has demonstrated improved cognitive outcomes in patients with HA-WBRT and memantine compared to traditional WBRT with memantine [29•]. Several randomized trials are presently comparing this treatment to SRS with regards to clinical efficacy, cost effectiveness, and cognitive function [28, 39].

## Surgery

The role of surgery for brain metastases has generally been in the setting of large or symptomatic disease. In these cases, surgical removal may improve both survival and functional independence [40]. This must be weighed against possible surgical risks, as performance status remains a significant predictor of brain metastasis patient prognosis [41]. While craniotomy remains the gold standard surgical technique, the surgical toolset has expanded over time to include laser interstitial thermotherapy (LITT) which may be able to access deeper lesions. Moreover, in cases where prior SRS has already been delivered to the operative bed, use of BCNU wafers offers a non-radiotherapeutic adjuvant option to improve local control within the resection cavity.

Much of the surgical literature was derived from a time period in which patients presented with large and symptomatic brain metastases. There has been a stage migration of brain metastasis patients in the past 2 decades as brain metastases are now more commonly diagnosed with staging MRI as smaller asymptomatic lesions. As such, it is important to interpret data from surgical series in the proper context which has generally been that of larger and symptomatic brain metastases.

Craniotomy (resection/debulking) relative contraindications: eloquent brain, deep lesions, multiple lesions, brainstem lesions, medically infirm, uncontrolled (or end stage) systemic disease, anticoagulant use (needs to be discontinued/reversed)Complications: infection, wound healing complications, hemorrhage, stroke, seizure, transient/permanent neurologic deficits, hydrocephalus, cerebrospinal fluid complications, iatrogenic leptomeningeal seeding, thromboembolic events.

Special points: Craniotomy can be performed to remove larger tumors (> 3 cm), to acquire pathologic confirmation of cancer and/or relevant mutations/treatment targets, and to remove lesions causing significant mass effect. Without adjuvant treatment the local recurrence rate may be up to 50% highlighting the need for multidisciplinary care. In the setting of recurrence or radiation necrosis after SRS, craniotomy can effectively treat these post-treatment sequela.

### BCNU wafer placement

Contraindication: resection cavity contiguous with ventricle.

Complications: infection, seizure, wound healing complications, hydrocephalus.

Special points: BCNU wafers can be placed in conjunction with a craniotomy in the setting of pathologically proven recurrent brain metastasis that has previously been treated with SRS or WBRT [42]. The BCNU is delivered at a very high dose to the local resection cavity, the highest risk location of tumor recurrence after resection.

### Laser interstitial thermotherapy (LITT)

Contraindication: tumor greater than 3 cm, tumor in primary motor cortex or Broca’s/Wernicke’s regions, presence of non-MRI compatible implant.

Complications: hemorrhage, seizure, transient/permanent neurologic deficits, post-treatment edema.

Special points: LITT can be performed for lesions smaller than 3 cm that are accessible by a probe using the same trajectory as a biopsy tract. In general, a biopsy is performed during the same procedure as the LITT. A clinically useful indication is the diagnosis and treatment of post-SRS imaging changes as both tumor progression [43] and radiation necrosis [44] can be effectively managed with LITT.

## Assistive devices

Imaging plays an important role in both diagnosis of brain metastases and response assessment after treatment. While diagnosis will also include pathologic confirmation of either the brain lesion or some other metastatic or primary lesion, response assessment after treatment may rely upon serial imaging for some period of time before a decision is made to either biopsy or offer further treatment. Of note, brain metastases that are treated with SRS can have imaging changes that can occur months or even years after the original treatment, and these changes may represent either tumor recurrence or treatment-related changes [45], Conventional MRI is often unreliable attempting to distinguish these entities, and therefore novel sequences or ancillary imaging may be required to aid in making this diagnosis.

### Magnetic resonance imaging (MRI)

Usage: Diagnosis and also for response assessment and screening for new brain metastases after treatment. MRI can also be used for preoperative assessment.

Special points: Some pulse sequences may have specific uses in the management of brain metastases. Fluid attenuated inversion recovery (FLAIR) imaging can assess for peritumoral edema. Functional magnetic resonance imaging and diffusion tensor imaging can be used to identify eloquent cortex and key white matter tracts to help with preoperative planning and avoidance of crucial anatomic structures [46, 47]. Perfusion-weighted imaging can be used to differentiate between treatment-related changes after radiation vs. tumor progression [48].

### Magnetic resonance spectroscopy

Usage: Comparison of chemical composition of a normal region of the brain to an abnormal region to assist with response assessment after SRS.

Special points: MR spectroscopy is often used as a non-invasive means of helping to determine tumor progression vs. treatment-related changes after radiation [49].

### Positron emission tomography (PET)

Usage: Use of radioactive tracers to assess for regional uptake within the brain to assist with response assessment after SRS.

Special points: F18 amino acid PET has been used as a non-invasive means of helping to determine tumor progression vs. treatment-related changes after radiation [50, 51]. In general, these tracers benefit from low background physiologic accumulation in the brain, enhancing tumor to background contrast and include 18F fluciclovine, 18F fluoroethyltyrosine, 11C methionine, and 18F DOPA. Traditional F18 fluorodeoxyglucose has also been utilized to some success [51].

## Emerging therapies

At present, the combination of early diagnosis, radiotherapy, surgery, and systemic therapies has improved brain metastasis outcomes compared to the standard management just two decades ago. However, in spite of these advancements, nearly 20% of patients will still die of brain metastases [13]. Novel therapies for brain metastases can be categorized into either salvage therapies that have progressed after multiple local therapies, adjuvant therapies meant to decrease the likelihood of further seeding of the brain, or radiosensitizers which attempt to improve the effectiveness of radiotherapy in the setting of metastases that are more likely to recur (e.g., larger lesions).

### Tumor treating fields (TTF)

Contraindications: skull defects, pre-existing deep brain stimulator.

Complications: scalp irritation, headaches.

Special points: A phase III trial is being conducted for the use of TTF in conjunction with SRS for patients with brain metastases from NSCLC [52].

### Surgically targeted radiation therapy (STaRT)

Contraindication: exceeded cumulative radiation tolerances, cavity greater than 5 cm, seed location < 5 mm from optic chiasm or brainstem, hypersensitivity to bovine-derived materials.

Complications: CSF leak, infection, delayed hemorrhage, adhesions, seizure, radiation necrosis.

Special points: Cs-131 seeds embedded in collagen tiles are a form of permanent brachytherapy placed at the time of metastasectomy. The theoretical benefit of STaRT is the immediate treatment of the surgical cavity compared to post-operative SRS which is generally delivered several weeks after surgery. There may be dosimetric advantages over repeat SRS to previously irradiated cavities [53].

### MR-guided focused ultrasound (MRgFUS)

Contraindications: non-MRI compatible implant, insufficient skull thickness.

Complications: peritumoral edema, thrombotic event, hemorrhage.

Special points: MRgFUS is being assessed as a treatment option for brain metastases because of its ability to disrupt the blood brain barrier and allow for entry of systemically administered agents that otherwise may not cross the blood brain barrier [54].

### Genomically-guided treatment

Contraindications: specific to individual agents used.

Complications: specific to individual agents used.

Special points: The Alliance A071701 study is investigating the use of genomically-guided treatment for progressive brain metastases [55]. Next-generation sequencing analysis will identify targetable mutations in prior brain metastasis tissue, and patients are treated based on this analysis. This technique may hold potential in the treatment of brain metastases that have failed multiple local therapies or with diffuse intracranial failure.

### Dendritic cell vaccines

Contraindications: immunosuppression.

Complications: flu-like symptoms, injection site reactions.

Special points: Dendritic cell vaccines can be developed to induce an immune response to a specific antigen (presently being investigated for Her2/Her3) and thus allowing for a more targeted cancer immune response. In combination with an immune checkpoint inhibitor, such as pembrolizumab, the goal is to induce a robust, targeted immune response that may more effectively shrink or eliminate tumors in the brain [56]. At this time, this approach is being assessed in a phase II study.

### Activation and guidance of irradiation by X-ray (AGuIX) gadolinium-based nanoparticles

Contraindications: renal insufficiency, non-MRI compatible implant, general radiotherapy contraindications.

Complications: headache, nausea, asthenia, sepsis (rare).

Special points: Gadolinium-based nanoparticles are radiosensitizing and preferentially retained in tumors versus normal tissues, allowing for the potential to provide equivalent disease control with dose reduction [57]. A Phase II prospective randomized controlled trial assessing whether AGuIX gadolinium-based nanoparticles with SRS are more effective than SRS alone for treating brain metastases is presently underway [58].
